# How are clinical exercise physiology postgraduate courses taught and assessed in the UK? A multimethod qualitative exploration

**DOI:** 10.1136/bmjopen-2025-099240

**Published:** 2025-05-27

**Authors:** Anthony Crozier, Gemma Miller, Lee Graves, Ellen A Dawson, Connor Osin, Ian Sadler, Louise H Naylor, Daniel J Green, Christopher David Askew, Helen Jones

**Affiliations:** 1 Sport and Exercise Science, Liverpool John Moores University Faculty of Science, Liverpool, UK; 2 Liverpool John Moores University, Liverpool, UK; 3 The University of Western Australia, Perth, Western Australia, Australia; 4 School of Sport Science, Exercise and Health, The University of Western Australia, Crawley, Western Australia, Australia; 5 Research Institute for Sport and Exercise Sciences, Liverpool John Moores University, Merseyside, UK; 6 School of Health, University of the Sunshine Coast, Sippy Downs, Queensland, Australia; 7 Sport and Exercise Sciences, Liverpool John Moores University, Liverpool, Merseyside, UK

**Keywords:** EDUCATION & TRAINING (see Medical Education & Training), Exercise, REHABILITATION MEDICINE

## Abstract

**Abstract:**

**Introduction:**

Development of clinical skills in areas, such as exercise risk stratification, testing, prescription, monitoring and outcome assessment, is vital for patient safety and clinical effectiveness in clinical exercise physiology (CEP). This study explored how current CEP courses are being taught and assessed and to identify potential best practice recommendations from a variety of stakeholders

**Methods:**

Qualitative methods were employed to explore the thoughts of CEPs, academics and current students regarding the teaching and assessment of CEPs in the UK. Research design involved (1) semistructured interviews with students (n=16) and (2) focus groups with academics (n=8) and CEP (n=5) stakeholders. Data obtained were audio recorded using a portable Dictaphone and transcribed verbatim, then thematically analysed manually.

**Results:**

Three themes: (1) in situ learning/real-world practice (working with patients and specialist practitioners); (2) programme design (scaffold learning and integrated modules) and (3) teaching approach (simulated learning and research competency) were generated concerning teaching methods and approaches across CEP postgraduate degrees. The current use of simulated tasks for the delivery of taught content was identified as lacking effectiveness, with clinical placements identified as being the most important source of knowledge and skill attainment due to the real-world exposure to patients and practitioners. Clinical placements and simulated learning were recognised as the two main methods of problem-based learning used to develop student knowledge, skills and competency to practice. Two themes (placement tariffs/assessors in situ and role play/simulation) were identified for the assessment of students.

**Conclusion:**

Clinical placements remain the optimal method for developing the knowledge, skills and competency to practice for student CEPs. However, suitable placements remain limited, and novel approaches such as university-led exercise services require consideration for student competency development. A standardised and accredited training pathway from undergraduate through to postgraduate level should be explored to allow student competency to be developed over a longer period, to enhance knowledge, skills and competency on graduation and registration.

**STRENGTHS AND LIMITATIONS OF THIS STUDY**

- The semistructured interviews and focus group participants consisted of academics, clinical exercise physiology (CEPs) and students with experience in teaching and assessments within a postgraduate master’s degree in CEP.
- The sample size was identified as sufficient for focus group data collection.
- The generalisability of the findings beyond the sample is limited regarding student insight as they were recruited from one institution only.

## Introduction

 The purpose of teaching is to facilitate learning and the development of knowledge and skills required to practice in society.^1^ Learning concerns the way in which we perceive and understand the world; it is about establishing meaning and in a wider context may involve mastering intellectual principles, recalling information, understanding methods, techniques and approaches and debating ideas.^2 3^ In the field of clinical exercise physiology (CEP), the development of clinical skills pertaining to exercise risk stratification, testing, prescription, monitoring and outcome assessment is vital as they can have profound implications on patient safety and clinical efficacy.^1 4^ The challenge for those responsible for educating and training CEPs at university level is how best to ensure that students develop the requisite theoretical knowledge alongside the practical professional competencies they require to be able to practice in complex clinical settings.^5^

There is a substantial body of evidence which indicates that student learning is maximised through peer-on-peer cooperative working.^6^,^8^ Such practice builds on foundational knowledge and enables learners to refine their understanding as they gain experience in dealing with new situations.^6^,^8^ Peer interaction within CEP curricula is frequently supported by blended learning, which is a broad term used to describe a combination of face-to-face teaching with technology-mediated instruction.^8 9^ As an extension of peer interaction and blended learning within course delivery, clinical placements allow students to observe and take part in clinical practice, forming an essential criterion in accredited CEP master’s degrees.^4^ Internationally (including in the UK) accredited CEP courses or equivalent include the application of theory in a practice-based setting via clinical work placements, with an expectation that this will assist in the development of competent work-ready graduates.^4^ Work-based learning situations are inherently variable, unpredictable, sometimes brief and frequently high risk, providing students with unrivalled exposure to the role of a CEP.^2^,^4^ The sparse availability of placements during the maturation of the profession, however, has potentially forced universities to rethink their individual curriculum design and assessment practices for CEP degrees and increase the need for innovative strategies to provide clinical learning opportunities.^4^ Consequently, there is a greater need to understand and simulate effective learning environments within CEP teaching, alongside a need to devise realistic assessments that will replicate clinical workplace settings and test graduate competency.^4 8 9^

Two methods used in blended learning are ‘flipped classrooms’ and problem-based learning (PBL), which includes simulated learning environments and activities.^8 10^ Flipped classroom learning, where lectures and other theoretical content are made available to students in advance, often in an online format, allows students to study/research theoretical concepts in their own time (asynchronous learning), individually or in a group format.^10 11^ Consequently, face-to-face teaching time can be spent undergoing interactive activities specific to practical skill implementation, such as exercise delivery for CEPs.^12 13^ Similarly, emerging from the need to emphasise active learner engagement and the construction of student knowledge through individualised research, PBL focuses on teamwork and communication and is widely adopted in healthcare education settings, including CEP courses.^14 15^ Recognised as one of the most innovative instructional methods, PBL has been integrated within CEP teaching in a variety of ways, such as case studies, research evidence gathering or real-world practical simulations.^9^ PBL originated in response to the criticism that traditional teaching and learning methods failed to prepare medical students for problem-solving in clinical settings.^16 17^ The ability to embed CEP students’ learning processes in real-life scenarios, rather than forcing engagement through context-free problems, has allowed PBL to assist in bridging the gap in practical skill application during CEP courses.^16^ Practical skills are particularly significant in scientifically based disciplines (such as CEP) where active and authentic student participation is central to the development of understanding phenomena, asking questions, constructing explanations, comparing findings with current scientific knowledge and then communicating outcomes to peers, all of which is vital for optimal patient care.^4^

Since the creation of the Clinical Exercise Physiology UK (CEP-UK) curriculum framework and scope of practice, in conjunction with the Academy for Healthcare Science (AHCS) standards of proficiency and good scientific practice documents in the UK, there has been no recommended best practice evidence regarding effective teaching and assessment of students undertaking the newly accredited CEP master’s degree. Therefore, this study aimed to identify how current CEP courses are being taught and assessed in the UK and to identify potential best practice recommendations from a variety of stakeholders.

## Methods

### Research design

Multiple qualitative methods were employed to explore the participants' perspectives both individually and collectively.^18^ This qualitative multimethod approach, which included data triangulation and large participant numbers, was employed to enhance collective discussions and interpret the consensus of opinions of stakeholders that infrequently interact (CEPs and academics) and to reduce potential social desirability and bias from stakeholder perspectives that interact daily (students).^19^ Our multimethods research design involved (1) semi-structured interviews with students from one AHCS-accredited master’s degree and (2) focus groups with academic and CEP stakeholders. This design allowed the generation of rich data and key themes that could inform future teaching and assessment methods across CEP master’s courses in the UK.^20^ Ethical approval was obtained from Liverpool John Moores University Research Ethics Committee (ref: 22/SPS/009).

### Participants and recruitment

The participants were identified using various recruitment strategies. Purposive and snowball sampling by direct contact with academics identified from university websites, and healthcare professionals identified through databases of exercise rehabilitation services across the UK^21^ were the primary methods. Student recruitment was completed via public advertisement on social media (eg, X) and snowball sampling. The final sample included academics from UK institutions (n=8), registered AHCS CEPs (n=5) (table 1) and master’s students from a current accredited CEP university course in the UK (n=16) (table 2). Demographic information (name, email, occupation, current workplace setting, gender, ethnicity, years of experience in a clinical exercise-related role, if they were AHCS registered or not, and whether they worked in the UK) was collected and used to identify participants who were eligible to participate. No one formally declined or stated any reasons for not taking part. Written informed consent was gained prior to study commencement, and a reimbursement to student participants was provided via a £10 shopping voucher for taking part in interviews. Given the small sample size, it is important to note that “data saturation” or “data adequacy” could be validated as no new themes were identified when analysing the final few transcripts.^22 23^

**Table 1:** The focus group participant demographic information

Stakeholder	Gender	Ethnicity	Years of experience in a clinical exercise-related job role	AHCS registered	Work in the UK
Academics (n=8)Registered CEPs in clinical practice (n=5)	Male (n=7)Female (n=6)	White (n=13)	0–5 years (n=1)6–10 years (n=5)11–15 years (n=4)16–20 years (n=1)	Yes (n=12)No (n=1)	Yes (n=13)

AHCS, Academy of Healthcare Science; CEP, clinical exercise physiology.

**Table 2:** The semistructured interview participant demographic information

Stakeholder	Gender	Ethnicity	UK-based university
MSc students (n=16)	Male (n=6)Female (n=10)	White (n=15)Asian (n=1)	Yes (n=16)

AHCS, Academy of Healthcare Science.

### Patient and public involvement

Patient and public involvement was completed during the ethics application, with people who had accessed previous interventions with CEPs asked whether it was worthwhile to explore how postgraduate CEP courses were taught and assessed. All responses recognised the work as valuable for the development of the profession, with the research methods identified as suitable.

### Eligibility criteria

The participants included were those who fulfilled one of the criteria listed below:

- Current student within a CEP master’s level course in the UK.
- Work/previous experience as a healthcare professional within the field of clinical exercise in the UK.
- Work/previous experience in an academic role within the field of clinical exercise in the UK.

### Semistructured interviews

The semistructured interview guide (see online supplemental additional file 1) was developed based on the questions deemed most pertinent to understand how a master’s course was currently taught (eg, what factors were important for optimal teaching, learning and assessment and which areas potentially needed improvement). The interview questions were based on the CEP-UK curriculum framework^4^ and used the six components (pathophysiology and clinical management, screening and risk assessment, assessment of health status and functional capacity, design of exercise interventions, exercise delivery and implementation, and behaviour change and communication) required within an accredited master’s degree to guide participants. Within this, we maintained a broad focus and used open questions to allow stakeholders to respond to the issues they deemed most important. Interviews (n=16) were conducted on an individual basis by CO (alone) with the student participants. Interviews took place in a private office within the university campus, lasting 24 min on average (ranging from 15 minutes to 43 minutes). Pilot interviews were conducted by CO with three independent researcher peers prior to study commencement to enhance credibility and refine interview questions where necessary.^24^ Prompts and probes were used during interviews to elicit more detailed responses from participants, where appropriate.^20^ At the end of each interview, a brief verbal summary was provided by the researcher to clarify the main points and allow participants to add further information.^25^

### Focus groups

The three focus groups (a mixture of academics and registered CEPs in each) were conducted by the first author online via Microsoft Teams. The focus groups lasted 70 min on average (ranging from 59 min to 90 min) and were based on the same semistructured interview guide. Spontaneous conversation was encouraged by the facilitator, with the peers able to discuss and challenge opinions if they wished.^26^ Participants were semifamiliar with each other, having previously met in some cases due to previous collaborative working across the industry, meaning that the interactions felt honest with reduced social desirability or bias.^26^ Clarification of information was sought during the questioning process to ensure participants were able to expand on each other’s opinion and summarise the information provided.^26^

### Data analysis

Data obtained through the focus group and semistructured interviews were audio recorded using a portable Dictaphone and transcribed verbatim. Data were manually thematically analysed using recommendations such as data familiarisation, generating initial themes, coding and finalising patterns of data with shared interpretations underpinned by an overarching concept and writing up using data extracts interspersed with researcher insights and interpretations.^27^ Flexibility in analysis was driven by both the prevalence (number of speakers articulating the theme) and the importance placed on information.^27^ Primary analysis was conducted by the first author. Frequent debriefing sessions with the research team to discuss, challenge and reframe the thematic structure were undertaken.^28 29^

## Results

Interview and focus group data were combined to provide a multistakeholder insight into the teaching, learning and assessment methods being experienced by students on the higher education CEP programmes. Table 3 illustrates the themes and subthemes identified during the analysis of teaching supported by verbatim quotes.

**Table 3:** Optimal methods of teaching and assessment as identified by participants

Optimal teaching /assessment	Themes /subthemes	Illustrative quotes
Teaching	In situ learning/real-world practice	
	Working with patients	“Patient access in a live setting allows for authentic problem-based learning to take place.” (AC2)
	Access to specialist practitioners	“We need to use practitioners with experience of working with specific populations…currency in the field but also an understanding of the practicalities of the role rather than what we perceive or how being a CEP works in theory…maybe the challenges they need to overcome.” (AC1)
	Programme design	
	Scaffolded learning	“I think it’s important to look at standardising some of the content taught at undergraduate…build the skill levels in undergraduate programmes to make the transition into postgraduate learning easier” (CEP2)
	Integrated modules	“Rather than compartmentalising content by modules…having a case study approach where you're looking at the physiology and theory of one or a couple of conditions together helps student understanding…then factor in the behavioural change and psychological impact, the exercise design and delivery…the patient experience all at once, I think this is a better way to learn” (AC5)
	Teaching approach	
	Simulated learning	“We use simulation…given the amount of placement time our students need and how competitive it is for clinic time, I think some of that time could be spent doing some simulation…essentially, students are given a scenario, a case study which they work through” (AC4)
	Research competency	“Flipped learning is one way to implement research skills but it can be hard to enforce…(students) need to demonstrate the ability to go away and gather information…learning independently…at masters level you should be expected to go away and research, learn and understand” (CEP3)
Assessment	Placement tariffs and assessor in situ	“Placement-based assessment would be ideal…We have a practice education facilitator who takes charge of all the clinical placements for the nurses and the AHPs, they would have assessor training every so often, so I guess it would be drilling down to know those that are going be the assessors in the clinical placements…having some continuity across that level of what you're agreeing is acceptable / what’s not acceptable” (CEP1)
	Role play and simulation	“In our practical exams and in preparation for the practical exams, student actors come in, we brief them and give a script with limitations such as wheelchairs, vision goggles, weighted vests, so they can emulate some of those physical constraints of patients, then we get the students to think on the spot for their exercise delivery” (AC3).

### Teaching

#### In situ learning and real-world practice

Academics and CEPs identified that having real-world exposure to patients as part of the teaching practice within university settings would enhance student skillsets, specifically their ability to communicate across diverse populations (eg, age, ethnicity and socio-economic backgrounds). Students recognised that interacting with peers in role-play scenarios was not effective or comparable when trying to develop practical application of theoretical skills in crucial competencies such as behaviour change and exercise delivery:

…I think (I’d like) more opportunities to get my hands on patients in practice…you know, prescribing exercise for real…it’s not easy, there’s a lot to take into consideration…that would be beneficial (Student 15)

There was, however, an acknowledgement that accessing patients during practical sessions in the university, although optimal, would be challenging:

…I think there’s a lot of a lot of barriers around accessing patients…transport, getting people in on time, particularly if they do have medical conditions, making sure they’re in a healthy state to come in on the day, parking, navigating the building which is often a rabbit warren…I think that there’s a lot of structural barriers as well as potentially a lot of physical barriers…then just trying to find patients who are willing to come in and are happy to talk to students…who pays for their time, can we? Can we incentivise patients to come and talk through? Is that ethical? (Academic 3)

It was highlighted by all participants that patient interaction increased during work placement; however, limitations were expressed concerning the duration (length of time in placements was too short) and effectiveness (suitability as not all services were delivering exercise) of placements by all stakeholders. Academics and CEPs identified that authentic lived experiences should be at the forefront of teaching delivery. During discussions, CEPs stressed that having current practitioners reflect on how they overcome daily challenges, either patient-related or environmental constraints in the role, would provide students with a true representation and clear expectation of the career pathway they were undertaking:

…somebody that has got experience of working with within that field… students really benefit from speaking to people who have worked in (or currently work in) the field…academics are great at certain things, theory, pathophysiology and so on, but for hands on exercise delivery current CEPs should be used if possible (Academic 2)

Work placements were identified as a facilitator for increasing contact time with practitioners. All participants recognised that observing CEPs ‘in action’ provided an in situ understanding of complex situations, such as communication skills, including how to ask questions to obtain key information:

…placements are key when we talk about transferable skills learnt from other professionals…observational activities, signs, symptoms, asking the right questions about maybe some of the more, not obscure, but kind of unusual signs and symptoms…getting the students drilling into that awareness of transferability of skills from one area of your role to another (Academic 1)

#### Programme design

Student competency levels on entry into a CEP master’s course were widely discussed by participants with recognition that increased levels of standardised prior learning are paramount to facilitate successful student understanding of the course content in areas, such as exercise prescription and delivery:

I think it’s important to look at standardising some of the content taught at undergraduate level…my experience is that all universities are different even with the same named degree…I guess starting from a more level platform you can assume more baseline knowledge…there is a difference between students graduating from different universities with apparently the same degree but with very different skills or depth of knowledge… everyone should be on the same level when they come up to level seven otherwise you're teaching a masters with very different abilities which is tricky. (CEP2)

Significantly, students shared concerns about being ‘set up to fail’ (Student 16) due to a lack of preparation in key areas such as exercise screening and prescription and delivery, acknowledging that they had ‘low talent’ (Student 15) in these areas and ‘would have benefitted if some of the practical content had been taught at undergraduate level’ (Student 13). The possibility of undergraduate placements was identified as a method of preparing students for the level of competency required in the master’s courses:

…offering a second year industry placement in undergraduate degrees is so beneficial. When they come back, it’s quite phenomenal to see the transformation, but then in their final year you could build on this…I think the key thing is teaching skills rather than teaching content…this will better prepare them for postgraduate studies or industry roles. I think undergraduate programmes need to be more flexible and more transferable skills focused (Academic 3)

Theoretical content attainment in modules such as pathophysiology did not register any concern compared with the practical, patient-facing elements, where a potential shortfall in competency was frequently identified. All stakeholders identified that having an integrated approach to learning medical conditions, rather than a module by module, isolated teaching approach would be beneficial:

Rather than compartmentalising content by module…having a case study approach where you're looking at the physiology and theory, but also in terms of the behavioural change, the psychological impact…understanding the whole picture and having a full patient experience all at once is most beneficial…you need to link it all together (Academic 5)

Conversely, one consideration was highlighted around multimorbid patients and how teaching could begin to combine conditions through the learning process to ensure graduates could optimally ‘work’ with patients that had a variety of medical conditions:

If you're following that patient journey, that linear cardiovascular journey for example, it doesn't take into account that multimorbidity…so when students do get into real world scenarios, they're going to be like, oh my God, I don't know how to prescribe for somebody who was cardiovascular and type two diabetes…so there’s something there about that linear journey that we're probably still missing…it could be a case of building up to complex multi-morbid case studies once an initial foundation is learnt in all of the main conditions such as cardiovascular. (Academic 6)

#### Teaching approach

Simulated learning, identified as a viable option by academics to increase student practical skills, was seen as the next best alternative to patient and practitioner observations and interactions. It was therefore recommended to be included in course curricula. The simulated learning environment was described in a variety of ways, such as peer-to-peer role play:

…given the amount of placement time our students need and how competitive it is for clinic time, students are given a scenario, a case study which they work through together (Academic 4).

Yet, students did not all share this opinion, with peer-on-peer simulated tasks being identified as unrealistic:

…doing it with each other (peer simulation of tasks) wasn’t realistic as we knew what to expect and what to do…there were no difficulties as no one wanted to make it hard for someone else, also it was hard to be serious and not switch off (Student 15).

Other methods related to modifiable equipment and ‘mock’ clinics:

…we've got ‘mock’ wards in one of our buildings, it’s exactly like a ward you see in a hospital…background noises, mannequins are there and we use actors to present the students with a scenario of cardiac patient for example… students go in to talk to them about cardiac rehab, that sort of phase one in hospital approach then we debrief and talk about it…students can go onto placement and be more prepared… (Academic 5)

More technological advancements were identified in the form of virtual reality, however, the feasibility of implementing them within teaching practice seemed challenging:

…there’s a lot of interest in virtual reality…we've invested quite heavily, but I think it’s in the very early stages…it could potentially bridge that gap and enable that real world authentic type learning…but it’s the cost up front…it’s quite expensive and you need the expertise of people that can run the simulations and then you need to programme the simulations (Academic 4)

In keeping with previous themes, the suggestions detailed how to upskill students in the practical aspects of the CEP role and then translate this into effective teaching practice with minimal concern regarding theoretical knowledge attainment. Embedding research skills within the teaching process was identified as challenging by the academics, specifically those who did not include a research project within their course curriculum. Notably, academics and CEPs highlighted that understanding research underpinned clinical practice and, without those skills, translation of information into areas such as exercise prescription could lead to ineffective patient care:

Demonstrating their ability to go away and gather information is important…learn independently…as much as we talk about the practical element, at Masters level you should be expected to go away and research…then be able to take that knowledge into a practical setting. (CEP3)

Ideas regarding how students would engage with research in the degree were widespread, with some focusing on condition-specific guideline exploration and others linking assessments and the production of a module-based project or presentation. Students discussed the possibility of more mini research tasks involving case studies and condition-specific exercise prescription:

I think that most of the activities we complete are similar, we use research skills in all of them, but the one we did this most in was when we had to find out everything about a condition then write a ‘mock’ programme card for one of the case studies in the module handbook…it made us work together as a group and find the information (Student 12).

Yet, no definitive agreement on the best way to embed research skills into the courses was reached during the sessions.

### Assessment

Similar to taught content recommendations, assessment processes that were debated primarily related to practical assessments rather than overarching theoretical content.

#### Placement tariffs/assessors in situ

Academics and CEPs acknowledged that the placement landscape within healthcare was complex due to its competitive nature, but more so for CEPs given its infancy as a healthcare profession. Stakeholders identified that CEP practitioners in services could act as assessors in situ given their expertise in day-to-day clinical practice. However, barriers such as training and standardisation, additional workload, feasibility due to the limited CEP workforce and a lack of financial support (tariff) when compared with other professions potentially restrict such practice:

It really depends on the service as they're so different wherever you go, and there’s no guarantee that you've got a CEP in place…it would be difficult to ask places for an assessment of competency…but given the right placement I'd like to think it’s possible, our service for instance, it could be realistic…the difficulty is standardisation…training of assessors in the assessment criteria and the service having capacity to undertake the extra workload…plus a financial incentive might help! (CEP5)

#### Role play/simulation

As acknowledged during taught content suggestions, role play and simulation were identified as methods of assessment which could be used internally, rather than relying on external collaborations such as placement providers. However, authenticity was a concern during the assessment of modules such as behaviour change, as it was recognised that peer-to-peer role play was ineffective and failed to examine students' ability to unearth the psychological support required by a ‘patient’ due to the staging/scripted nature of the interaction:

…role play is often all we have, but if students know the person in front of them it becomes hard to separate fact and fiction…it’s the same if it’s a mock patient, you know, an academic from the teaching cohort…the only time it works is if we bring patients in, maybe relatives or family / friends of the teaching staff, or teaching support officers who tend to be postdoctoral students, but this that can be equally challenging to organise (Academic 8).

In agreement, students recognised that ‘real-life’ assessments using patients external to the course felt more realistic, but at the same time more daunting given the ‘limited patient experience’ (Student 4) they experienced on the course up to that point.

### Discussion

This study aimed to identify how current CEP courses are being taught and assessed in the UK and to identify potential best practice recommendations from a variety of stakeholders. A consistent theme generated by all stakeholders centred around the need for increased exposure to real-world settings, inclusive of patients and practitioners (preferably CEPs). Internationally, clinical placements have been notoriously difficult to secure for CEPs, culminating in various countries (eg, Australia) creating novel ways to replicate real-world environments, for example ‘in-house’ clinics or simulated learning.^30^ Academic institutions, including some in the UK, have devised internal solutions to the limited placement options by utilising their own university facilities to run clinical exercise services. These services are frequently overseen by qualified CEPs (or equivalent) employed under University governance as academic or general staff, who retain registration with the accrediting body in that country (eg, AHCS in the UK) and provide support and education for students to deliver exercise interventions for the general public, across multiple long-term conditions. Such practice was identified by stakeholders as a potential solution to the difficulties in securing clinical placements through the provision of authentic PBL in the UK setting. A multitude of benefits for university-based exercise services have been reported in other countries, such as (1) an increased opportunity for students to actively engage with patients in clinical settings and therefore reducing the volume of external placements needed; (2) observation of, and ‘hands-on’ practical experience in a learning environment with real patients that removes the need for less effective peer-to-peer role play and (3) in situ assessment opportunities within CEP courses which can be facilitated by practitioners with real patients.^30^ This approach therefore has multiple virtues; it provides a readily accessible and highly supportive practicum setting for CEP education, it embeds experienced CEPs with students, and it is seen as an exemplar of University outreach in terms of service to the local community. Notably, stakeholders also recognised the crossover between teaching and assessment design that could occur by effective placement strategies such as internal clinics. Competency-based assessments completed by a practitioner during placements were identified as optimal, akin to previous research,^31 32^ yet previously acknowledged barriers became evident concerning increased clinical staff workloads, assessor training requirements, time costs and placement capacities. This solution, although most favourable, was still unrealistic currently in the UK, yet it was clear that developing novel pathways for students to obtain experience within clinical practice should be at the forefront of curriculum frameworks.

In conjunction with, or as a potential option to internal clinics, simulated learning as a form of PBL was proposed as a tool that could be explored both for teaching and assessment purposes. Widely employed in the formation of CEP lesson plans to augment the student learning experience, the simplest version of simulated learning was acknowledged as peer-to-peer role play. Yet, there was agreement between students and academics that this form of interaction was largely ineffective, further compounded by student recognition that it felt awkward and often resulted in an unproductive experience. Conversely, simulated learning has been deemed successful when non-peer-to-peer activities have been used in PBL.^30 32^ Using practising CEPs or equivalent with training in simulation activities to facilitate scenarios, alongside professional actors who retained experience in various medical and healthcare simulations, has seen favourable learning outcomes for students.^9 30 32^ This level of robustness when designing simulated learning tools has been successful in promoting student confidence in communication skills and positively impacted students’ motivation to learn and apply their skills in clinical practice via improved perceived competence.^33^ Thus, it is important to note that not all simulated learning is equal. In keeping with previous research and stakeholder experiences regarding ineffective experiences via peer-to-peer scenarios, it is vital that any simulated learning activities used in CEP teaching and assessments are as close to real-world scenarios as possible, to retain relevance and usefulness.^30 32^

Undergraduate degree standardisation and the ‘scaffolding’ of learning was recognised as an area of opportunity by stakeholders. Academics identified that a variation in taught content at undergraduate level contributed to gaps in student knowledge and skill levels on entering CEP masters courses. This was further highlighted by students acknowledging feeling a sense of apprehension in certain areas of the syllabus as they felt it was assumed knowledge (by lecturers) which they did not have. A standardised and accredited curriculum for undergraduate degrees should therefore be considered to create a succinct transition into postgraduate learning. A potential solution to the disparate levels of training at undergraduate level has been seen in Australia via Exercise and Sport Science Australia (ESSA).^34^ ESSA accredits multiple curriculum frameworks, one for undergraduate courses (students can work with apparently healthy individuals with low level risk factors such as age and weight), one for postgraduate courses (students can work with high-risk clinical populations), and a longer 4-year pathway that combines the two and ensures that core competencies are taught and assessed throughout the learning pathway.^34^ Further, a similar framework in the UK, whereby no clinical placement hours are currently obtained at undergraduate level, was identified as a method of enhancing student competency levels while providing additional time for reflection regarding clinical practice in real-world settings, an area also highlighted as essential in previous research.^1^ One area of debate was the variety of methods being employed when embedding research skills into student activities and the uncertainty of how this should be assessed. Academics referenced that more guidance from the CEP-UK curriculum framework would be useful, specifically the areas in which research skills could be incorporated, for example, condition-specific exercise guideline presentations or reports.

### Strengths and limitations

The participants consisted of academics, registered CEPs, and current CEP master’s students who have all had experience of the teaching and assessment practice within CEP courses. The sample size was identified as sufficient for a multi-method qualitative study based on data saturation. The participants in this study were actively engaged in the field of CEP, which adds robustness to the evidence and provides clarity in the competency levels required of new graduates based on their experiences. However, the findings beyond the sample regarding student opinions are limited due to single institution recruitment.

### Conclusion

This study aimed to identify how current CEP courses are being taught and assessed in the UK and to identify potential best practice recommendations from a variety of stakeholders. Significantly, taught content currently utilises PBL as a primary method of student learning and engagement, yet the effectiveness of its delivery using simulated tasks is questionable in its current format, with further exploration into the training of educators in this area being required. Clinical placements remain the greatest source of knowledge and skill attainment due to the real-world exposure to patients and practitioners. However, suitable placements remain limited. Therefore, more novel ideas such as university-led exercise services require consideration for student competency development. Finally, a standardised and accredited training pathway from undergraduate through to postgraduate level was identified for future exploration to allow student competency to be developed over a longer period and therefore enhance knowledge, skills and competency on graduation and registration.

## Supplementary material

10.1136/bmjopen-2025-099240 online supplemental file 1

## Data Availability

Data are available upon reasonable request.
