# Supplementary material for: How are clinical exercise physiology postgraduate courses taught and assessed in the UK? A multimethod qualitative exploration
Source: BMJ Open. 2025 May 27;15(5):e099240. doi: 10.1136/bmjopen-2025-099240 (PMC12121592; doi:10.1136/bmjopen-2025-099240)
Supplement: online supplemental file 1 [file bmjopen-15-5-s001.docx]

**Focus group and semi-structured interview questions**

Based on your knowledge of clinical exercise physiology degree(s) in the UK and your clinical experience, what type(s) of learning activities are used with students to meet the following standards:

1. Understanding the relevant pathophysiology of chronic diseases (e.g., CVD, cancer, metabolic conditions)?
2. Be able to screen and risk assess patients in clinical settings?
3. Be able to design an exercise intervention for a variety long-term conditions and multi-morbidities (e.g., CVD and diabetes)?
4. Be able to demonstrate and deliver an exercise session/programme in clinical practice for a variety of long-term conditions and multi-morbidities?
5. Understand how to support and communicate with patients regarding behaviour change in real world settings?
6. How do we/can we include research to practice within module content (e.g., guidelines)
7. Are there any other types of learning activities would you recommend that all students should undertake as part of a CEP degree?
8. Which skills and attributes listed above require “real world” or authentic learning activities and which activities can be covered in the classroom e.g., through simulation?
9. Out of the activities you have mentioned, which do you feel have had the most impact in preparing students for a role as a CEP and why?

Based on your knowledge of clinical exercise physiology degree(s) in the UK and your clinical experience, what form(s) of assessment would be best suited to meet the following standards:

1. Understanding the relevant pathophysiology of chronic diseases (e.g., CVD, cancer, metabolic conditions)?
2. Screening and risk assessment of patients in clinical settings?
3. Designing an exercise intervention for a variety long-term conditions and multi-morbidities (e.g., CVD and diabetes)?
4. Demonstrating and delivering an exercise session/programme in clinical practice for a variety of long-term conditions and multi-morbidities?
5. Understanding how to support and communicate with patients regarding behaviour change in real world settings?
6. Please provide any additional information you feel relevant that has not been mentioned.
