# Supplementary material for: How are clinical exercise physiology postgraduate courses taught and assessed in the UK? A multimethod qualitative exploration
Source: BMJ Open. 2025 May 27;15(5):e099240. doi: 10.1136/bmjopen-2025-099240 (PMC12121592; doi:10.1136/bmjopen-2025-099240)
Supplement: Supplementary file 2 [file bmjopen-2025-099240.reviewer_comments.pdf]

PEER REVIEW HISTORY

BMJ Open publishes all reviews undertaken for accepted manuscripts. Reviewers are asked to complete a checklist review form and are provided with free text boxes to elaborate on their assessment. These free text comments are reproduced below.

ARTICLE DETAILS

Title (Provisional)

How are clinical exercise physiology postgraduate courses taught and assessed in the UK? A multi-method qualitative exploration

Authors

Crozier, Anthony; Miller, Gemma; Graves, Lee; Dawson , Ellen A; Osin, Connor; Sadler, Ian; Naylor, Louise H.; Green, Daniel J; Askew, Christopher David; Jones, Helen

---

VERSION 1 - REVIEW

**Reviewer**	**1**
**Name**	**Raymond, Jacqueline**
**Affiliation**	**The University of Sydney, Faculty of Health Sciences**
**Date**	**25-Mar-2025**
**COI**	**None**

Peer Review

Thank you for the invitation to review this paper. I found this paper interesting, and I think it has the potential to make a valuable contribution. I have provided my commentary below for the various sections of the paper. I hope the authors find these useful for increasing the quality of the manuscript. The main things that I was struggling with were (i) alignment between the study aim, the interview/focus group questions and the results, and (ii) how the reflexive thematic analysis approach was used. It will be helpful if the authors can provide more clarity on these.

Introduction

I found the second paragraph, which starts on line 17 of page 3 difficult to navigate. The paragraph starts out with sentences about peer-to-peer learning and blended learning and moves to sentences about clinical placements without an obvious reason for the switch. I wonder whether the authors could consider reviewing the ordering of information in the introduction and how the argument is being developed.

There are, at times, in-text citations that seem implausible when cross-referenced to the full citation in the reference list. For example, on page 5, reference number 21 is cited twice in the top paragraph for statements related to curriculum in CEP degrees, however reference 21 is about qualitative data analysis *(Smith, J. and J. Firth, Qualitative data analysis: the framework approach. Nurse researcher, 2011. 18(2))*. This is not the only example where the referencing doesn't seem to align. Could the authors please check that the references in the reference list are all correctly attributed to the statements in the text and update as required?

The aim as stated on page 5, lines 16-18 lacks clarity. Is the aim of the study to explore how current CEP courses are being taught and assessed and also how they align curriculum with the real-world requirements of a CEP in the UK? Or is the aim just the first part of the sentence with the part about aligning curriculum a potential application of the findings of the study?

Methods

It is noted that the researchers used semi-structured interviews for students but focus groups for academic and CEP stakeholders? Could the authors provide more information in the research design section on why the approaches were selected for the different participant types?

My preference is that the outcome of the recruitment process is presented in the results section because the final sample is a result of the recruitment process. However, this is just my preference and if the convention for the journal or the researchers is to position this information in the Methods, I will defer to that.

Regarding the four eligibility criteria, could the authors clarify whether potential participants needed to fulfill just one of these? Or all four? Or a combination of some?

One of the eligibility criteria states that participants needed to be a current student within a CEP master's level course. However, students from only one university were recruited. Why weren't students from other universities also targeted in the recruitment? Is it because there is only one university in the UK that offers such a course?

The questions in the semi-structured interview and focus group guide (except for Q6 and Q9) do not appear to align with the aim of the study. This may have something to do with the lack of clarity of the research question (see comment above), and once that is resolved, the alignment will be evident. However, for the moment, it appears that the questions are asking about types of learning activities and assessments that would best prepare students. Furthermore, page 7, line 16 says that the questions were developed

"*to understand how a master's course could be best taught*". This contrasts with the study aim of exploring how current CEP courses are being taught and assessed.

Page 9, line 3. Remove the hyphen from stake-holders.

How many focus groups were conducted and how many participants were in each group?

Data Analysis – why was reflexive data analysis considered the most appropriate analysis for this study? Could the authors provide a rationale for this approach? It is also my understanding that when using reflexive thematic analysis, it is important for the researchers to present their underlying philosophical positioning as that would influence how they interact with the data.

Data Analysis – the actual reflexive thematic analysis is described generically through a set of recommendations. What is meant by "patterns of shared meanings underpinned by a central concept" (page 10, line 5-6)? Could the authors provide more detail on the analytical procedures used in this study?

After looking at reference number 29, this doesn't seem to be the most appropriate Braun and Clarke reference for the process the authors followed. Could you please check if this is the most appropriate reference in this instance?

Results

Some themes seem quite 'descriptive' in that they are summarising participants' suggestions for optimizing teaching and assessment practices along with barriers for implementing these. Is that the intention and does this align with the reflexive thematic analysis approach?

There seems to be limited student voice in the results with most views (and supporting quotes) arising from the academic and practitioner stakeholders. Furthermore, most of the suggestions for optimizing teaching and assessment practices come from the academics and CEPs. Are the authors able to provide a more balanced representation across their results so that the reader can be assured that the themes reflect the breadth of participants involved?

Discussion

The discussion extends on several of the themes identified in the results.

Pg. 21, line 3. Exercise and Sports Science Australia should be Exercise & Sport Science Australia.

Pg 21, lines 4-7. This is not the only model. ESSA also accredits undergraduate (usually 4-year programs) at the level of exercise physiology which permit graduates to work with clinical populations.

Regarding the limitations, generalisability in terms of being able to draw conclusions about a larger population of students based on the sample in the study is not usually considered a limitation of qualitative research. The paper by Smith (2017) might be useful in considering whether generalisability is a limitation in the context of this study. *Smith B (2018). Generalizability in qualitative research: misunderstanding, opportunities and recommendations for the sport and exercise sciences. Qualitative Research in Sport, Exercise, and Health, 10(1): 137-149.*

It was noted that the conclusion referred to the study aim as identifying how courses are being taught and assessed and also identifying potential best practice recommendations. This aim is closer to what appears to have been done in this study and could serve as a guide for revising the aim in the introduction.

References

The formatting of the reference list needs to be addressed.

Reviewer 2

Name Farahmandnia , Hojjat

Affiliation Kerman University of Medical Sciences, Health in Disasters and Emergencies Research Center, Institute for Futures Studies in Health, Kerman University of Medical Sciences, Kerman, Iran.

Date 03-Apr-2025

COI None

This is an interesting paper and the content is very good but I suggest the paper can be improved in the following ways: Abstract

-Please correct all parts of the article according to the guidelines of the journal authors guideline In the methods section please bring year of performing of this study, sampling methods and data analysis methods -In the conclusion part, it is necessary to specify the researcher's proposal to improve the conditions and use of the beneficiaries

Introduction

Please bring the following items 1- Definition of the research problem 2- The magnitude and importance of the study variable 3- Expressing the necessity of conducting the study Finally, the practical purpose of the study should be stated. Methods

Please report the details of data analysis approach and rigorous and interviw questions

Discussion

In the discussion section, it is necessary y to compare the main results of the study with the results of other studies in this field. To strengthen the article, especially in the introduction and discussion section the following studies are suggested, please used and add to this manuscript references.

- The effect of bio ethical principles education on ethical principles

- Investigating the burden of disease dimensions (time-dependent, developmental, physical, social and emotional) among family caregivers with COVID-19 patients in Iran

Conclusion What are your suggestion for future studies? Best regards

---

VERSION 1 - AUTHOR RESPONSE

Reviewer 1 Comments	Author response
**Introduction**<br>I found the second paragraph, which starts on line 17 of page 3 difficult to navigate.	Thank you – we have split this sentence to enable a clearer level of understanding – page 3 lines 15-18
The paragraph starts out with sentences about peer-to-peer learning and blended learning and moves to sentences about clinical placements without an obvious reason for the switch. I wonder whether the authors could consider reviewing the ordering of information in the introduction and how the argument is being developed.	Thank you for this feedback. We have now altered the wording to better link the peer learning elements to the clinical placements – page 3 & 4
There are, at times, in-text citations that seem implausible when cross-referenced to the full citation in the reference list. For example, on page 5, reference number 21 is cited twice in the top paragraph for statements related to curriculum in CEP degrees, however reference 21 is about qualitative data analysis (Smith, J. and J. Firth, Qualitative data analysis: the framework approach. Nurse researcher, 2011. 18(2)). This is not the only example where the referencing doesn't seem to align. Could the authors please check that the references in the reference list are all correctly attributed to the statements in the text and update as required?	Thank you for noticing this. It appears some of the references were mixed when we have edited previous drafts – this has now been corrected throughout the manuscript.
The aim as stated on page 5, lines 16-18 lacks clarity. Is the aim of the study to explore how current CEP courses are being taught and assessed and also how they align curriculum with the real-world requirements of a CEP in the UK? Or is the aim just the first part of the sentence with the part about aligning curriculum a potential application of the findings of the study?	Thank you for identifying the disparity between the introduction and conclusion aims (below comment). We agree that the study aligns and aims to understand how CEP courses are taught & assessed with best practice recommendations being an outcome – this has been updated – page 5 lines 15-17
**Methods**<br>It is noted that the researchers used semi-structured interviews for students but focus groups for academic and CEP stakeholders? Could the authors provide more information in the research design section on why the approaches were selected for the different participant types?	We have added in some further information to explain that the collective thoughts and opinions of CEPs and Academics were

My preference is that the outcome of the recruitment process is presented in the results section because the final sample is a result of the recruitment process.<br>However, this is just my preference and if the convention for the journal or the researchers is to position this information in the Methods, I will defer to that.<br><br>Regarding the four eligibility criteria, could the authors clarify whether potential participants needed to fulfill just one of these? Or all four? Or a combination of some?<br><br>One of the eligibility criteria states that participants needed to be a current student within a CEP master's level course. However, students from only one university were recruited. Why weren't students from other universities also targeted in the recruitment? Is it because there is only one university in the UK that offers such a course?<br><br>The questions in the semi-structured interview and focus group guide (except for Q6 and Q9) do not appear to align with the aim of the study. This may have something to do with the lack of clarity of the research question (see comment above), and once that is resolved, the alignment will be evident. However, for the moment, it appears that the questions are asking about types of learning activities and assessments that would best prepare students.<br><br>Furthermore, page 7, line 16 says that the questions were developed "to understand how a master's course could be best taught". This contrasts with the study aim of exploring how current CEP courses are being taught and assessed.<br><br>Page 9, line 3. Remove the hyphen from stake-holders.<br><br>How many focus groups were conducted and how many participants were in each group?	valuable as they infrequently interact, unlike students who could in part some social desirability on each other in group situations page 5.<br><br><br><br><br>We appreciate your comments. However, we have left this with the other participant information and will await any request from the editor to alter if required.<br><br><br><br><br><br><br>We agree that clarification was needed and have updated the eligibility criteria as requested – page 7.<br><br><br><br><br><br>Thank you for recognizing this. At the time of the study only one university had accreditation for the CEP MSc degree in the UK, therefore, those students were used in this study. We have acknowledged that a broader range of students would have been ideal within the limitations section.<br><br><br><br><br>Thank you for the feedback. We have now clarified the aims of the study (see above) and feel that the questions used reflect the research aims.

Data Analysis – why was reflexive data analysis considered the most appropriate analysis for this study? Could the authors provide a rationale for this approach? It is also my understanding that when using reflexive thematic analysis, it is important for the researchers to present their underlying philosophical positioning as that would influence how they interact with the data.	Thank you for noticing this error – we have changed to \`were currently taught\` to reflect the aims page 8 line 3.<br><br>Thank you – removed.
Data Analysis – the actual reflexive thematic analysis is described generically through a set of recommendations. What is meant by "patterns of shared meanings underpinned by a central concept" (page 10, line 5-6)? Could the authors provide more detail on the analytical procedures used in this study?	Thank you – added for clarification page 8 line 21<br><br>Thank you for your feedback. We agree that a thematic approach rather than a reflexive thematic approach has been used due to the structured nature of the topic guide and descriptive nature of the data itself, therefore we have altered this section to reflect our findings. Page 9.
After looking at reference number 29, this doesn't seem to be the most appropriate Braun and Clarke reference for the process the authors followed. Could you please check if this is the most appropriate reference in this instance?	
Results<br>Some themes seem quite 'descriptive' in that they are summarising participants' suggestions for optimizing teaching and assessment practices along with barriers for implementing these. Is that the intention and does this align with the reflexive thematic analysis approach?	Thank you for this feedback. We have adjusted as above.
There seems to be limited student voice in the results with most views (and supporting quotes) arising from the academic and practitioner stakeholders. Furthermore, most of the suggestions for optimizing teaching and assessment practices come from the academics and CEPs. Are the authors able to provide a more balanced representation across their results so that the reader can be assured that the themes reflect the breadth of participants involved?	Thank you for the feedback – we have updated the reference.
Discussion	Thank you for this feedback. As explained above we agree that a more descriptive set of themes have been generated and therefore adjusted the framing of the study analysis.

The discussion extends on several of the themes identified in the results.<br>Pg. 21, line 3. Exercise and Sports Science Australia should be Exercise & Sport Science Australia.	
Pg 21, lines 4-7. This is not the only model. ESSA also accredits undergraduate (usually 4-year programs) at the level of exercise physiology which permit graduates to work with clinical populations.	Thank you for this feedback. As previously noted, students were limited to one institution (noted in limitations) and had fairly limited opinions. However we have added in some more quotes to enhance the student voice and provide a more balanced discussion of both teaching and assessing where possible.
Regarding the limitations, generalisability in terms of being able to draw conclusions about a larger population of students based on the sample in the study is not usually considered a limitation of qualitative research. The paper by Smith (2017) might be useful in considering whether generalisability is a limitation in the context of this study. Smith B (2018). Generalizability in qualitative research: misunderstanding, opportunities and recommendations for the sport and exercise sciences. Qualitative Research in Sport, Exercise, and Health, 10(1): 137-149.	Thank you for this feedback – corrected.
It was noted that the conclusion referred to the study aim as identifying how courses are being taught and assessed and also identifying potential best practice recommendations. This aim is closer to what appears to have been done in this study and could serve as a guide for revising the aim in the introduction.	Thank you for this feedback – we have added in the 4-year pathway qualification. Page 21 lines 19-23.
References<br>The formatting of the reference list needs to be addressed	Thank you for this feedback. We agree that generalizability is usually for quantitative work, therefore, have re-worded to still highlight that only one site was used – page 22 lines 15-16.

	Addressed above<br><br>Thank you. We adjusted the reference list with corrections.
**Reviewer 2 Comments**	**Author response**
Abstract<br>-Please correct all parts of the article according to the guidelines of the journal authors guideline In the methods section please bring year of performing of this study, sampling methods and data analysis methods -In the conclusion part, it is necessary to specify the researcher's proposal to improve the conditions and use of the beneficiaries<br><br>Introduction<br>Please bring the following items 1- Definition of the research problem 2- The magnitude and importance of the study variable 3- Expressing the necessity of conducting the study Finally, the practical purpose of the study should be stated.<br><br>Methods<br>Please report the details of data analysis approach and rigorous and interview questions<br><br>Discussion<br>In the discussion section, it is necessary y to compare the main results of the study with the results of other studies in this field. To strengthen the article, especially in the introduction and discussion section the	Thank you for your feedback. The sample sizes are now included in the methods with analysis modality – page 2.<br><br>Thank you for your feedback. These elements are now included in the introduction as requested pages 3-5.<br><br>Thank you for your feedback. Additional information has been added as highlighted re: data analysis. Interview questions can be found in the additional file 1.<br><br>Thank you for your feedback. However, we do not feel that either of the papers cited

**following studies are suggested, please used and add to this manuscript references.<br>- The effect of bio ethical principles education on ethical principles<br>- Investigating the burden of disease dimensions (time-dependent, developmental, physical, social and emotional) among family caregivers with COVID-19 patients in Iran<br><br>Conclusion<br><br>What are your suggestion for future studies?<br>Best regards**	**here are relevant to this current study given this is specific to clinical exercise physiologist teaching and learning in university settings in the UK.<br><br>Thank you for your feedback. The recommendations are made regarding the development of a new training pathway from undergraduate to postgraduate study – page 23.**
